# Consumer knowledge and motivations for consumption of fermented foods

**DOI:** 10.3389/fmicb.2026.1789825

**Published:** 2026-04-10

**Authors:** Melanie Hanlon, Wannes Van Beeck, Lei Wei, Isabella Tosta, Ruofen Liao, Maria L. Marco, Erin DiCaprio

**Affiliations:** 1 Department of Food Science and Technology, University of California, Davis, Davis, CA, United States; 2 Department of Bioscience Engineering, University of Antwerp, Antwerp, Belgium

**Keywords:** consumers, fermented food, fruits, non-alcoholic, vegetables

## Abstract

Non-alcoholic fermented foods (FFs) are a popular food group with consumers; however limited studies exist evaluating the motivations for consuming FFs and the frequency of consumption. To begin to address this gap in knowledge, we developed an online survey to assess participant familiarity with different types of fermented products, determine consumption frequency, and gain insight into the motivation for consumption. A total of 751 participants completed the survey. Yogurt was the most frequently identified fermented food (*n* = 658; 87.62% of respondents). Participants reported consuming fermented cereal grains (*n* = 307; 46.17%), fruits and vegetables (*n* = 281; 42.26%), dairy products (*n* = 204; 39.70%), soy/rice products (*n* = 250; 37.60%) and fermented meats (*n* = 204; 30.68%). Reported daily consumption was highest for categories of fermented cereal and dairy products, compared to the other categories which typically were consumed on a weekly or monthly basis. The primary motivator for consumption was taste (*n* = 337; 50.68%) compared to health benefits (*n* = 235; 35.34%) and cultural reasons (*n* = 80; 12.03%). The most highly selected health benefits associated with FF consumption were “improved gut microbiome” (*n* = 513; 77.14%), “digestive benefits” (*n* = 508; 76.39%), and “probiotic” (*n* = 458; 68.87%). Participants associated health benefits with all fermented products listed in the survey. Therefore, consumers may assume that all fermented foods confer the same health benefits. The motivations for consumption (sensory attributes, health benefits, cultural reasons) did not vary when individuals were asked to respond for FFs as a broad category versus specifically for non-alcoholic, fermented fruits and vegetables. This suggests that consumers view FFs similarly regardless of the starting ingredients and fermentative process involved.

## 1 Introduction

Fermentation is a well-established food preservation practice enhancing flavor, shelf life and increasing safety, that is reliant upon growth of desired microbial species and/or communities. Although the necessity of using fermentation for food preservation has declined in many cultures, there has been a resurgence in general interest and consumption of these foods in Western-countries within the last decade (Dannic Drinks, 2022). The fermented food (FF) and beverage market is projected to grow at a compound annual growth rate (CAGR) of 6.43% from 2025 to 2030 (Mordor Intelligence, 2025). Understanding the interest in and desire for fermented foods can help grow this industry, based on data on the specific attributes of these foods that drive consumer demand.

FFs are defined as foods and beverages made through desired microbial growth and enzymatic conversions of food components (Marco et al., 2021). These foods are made using a diverse array of ingredients, including meats, milk, cereals, fruits and vegetables. Similarly, many different microbial species play central roles in the production of fermented foods and include microorganisms that rely on fermentation metabolism (for example lactic acid bacteria and yeast) as well as those which use respiration for energy conservation (for example molds and acetic acid bacteria). Depending on the ingredients and the desired food product, food fermentations can be completed in only a few hours (for example, yogurt) or take years to complete (for example, aged cheeses and wines). Therefore, fermented foods constitute a very broad food group which vary significantly based on the ingredients, environmental conditions, and microbial populations required for production.

FFs have been widely associated with health benefits including improved mental health, digestive health, and alleviation of metabolic and inflammatory disorders (Mukherjee et al., 2024; Caffrey et al., 2024). FFs are extremely complex foods that contain positive nutritional components associated with base ingredients, bio-active and other health promoting compounds produced as the result of fermentation, as well as in some products the live microorganisms responsible for fermentation (Wei et al., 2025). There is a growing body of evidence to support that the consumption of live non-pathogenic microorganisms has health promoting benefits. For example, certain strains of bacteria and yeast have been defined as probiotics, “live microorganisms that, when administered in adequate amounts, confer a health benefit to the host” (Hill et al., 2014). Moreover, recent evidence supports that ingestion of live non-pathogenic microorganisms, such as those find in fresh FFs, can have positive impacts on health as well (Marco et al., 2022; Hill et al., 2023). The gut microbiome is a diverse ecosystem of over a trillion microbes that supports extraction of nutrients from complex food components, modulates the immune system, helps protect against pathogen infection, among other important functions. While supplementation of the established gut microbiota with ingested live microorganisms is unlikely to result in colonization in the gut, these microbes provide similar health promoting effects over a short duration (Sanders and Hill, 2025).

FFs are well established in our global history, with recipes and knowledge being passed down in various regions through indigenous peoples (Hauptmann et al., 2025). While these traditional preparation methods remain important globally, the onset of accessible internet has allowed for rapid sharing of and access to personal recipes, advice, or guidelines. A recent meta-analysis found that of studies on consumer fermented food preference, sensory properties are the most widely evaluated and have been found to be primary driver for FF consumption (García-Barón et al., 2025). However, data on the general knowledge of consumers related to FFs broadly, consumption habits, as well as perceived sensory and health benefits, such as digestive benefits, improved gut microbiome, probiotic, prebiotic, improved nutritional value, immune system benefits is not widely available. This study aims to determine general awareness of different types of foods and beverages that are produced by fermentation. Moreover, the study aims to assess broadly the motivations for consumption of non-alcoholic FFs and compare those findings specifically to fermented fruits and vegetables as a category.

## 2 Materials and methods

### 2.1 Development of questionnaire

The consumer survey aimed to collect (a) the types of FFs people are consuming and (b) the motivation for consumption of FFs. The key research areas the survey aimed to address was: (1) consumer recognition and consumption of FFs; (2) primary motivators for purchasing and consuming FFs; (3) perception of health benefits conferred via the consumption of FFs; (4) influence of FF type on perception of conferred health benefits associated with consumption; (5) consumer interest specifically related to fresh fermented fruits and vegetables; (6) motivators for purchasing fresh fermented fruits and vegetables; (7) the prevalence of in-home fermentation of fruits and vegetables; (8) the types of resources individuals use to guide their home fermentation practices and (9) respondent demographics. Based on these research areas, a survey tool containing 26 questions was developed. To accompany the questions, pertinent background information defining FFs and fresh fermented fruits and vegetables were embedded within the questionnaire to provide background information to participants (Supplementary Materials, Supplementary Tables 1–5).

This analysis includes the questions in blocks 1, 2, 3, and 5. Analysis of question block 4 is excluded from this analysis. The first block of questions focused on the general category of fermented foods. Question (Q) 1 asked participants to identify the FFs from a provided list, all of which were fermented products. Q2 asked participants to self-report whether they consumed FFs other than fermented alcoholic beverages. In Q3, participants were asked to indicate whether they consumed FFs based on category (cereal grain, fruits and vegetables, dairy, soybean or rice, meats, or other) and provide the frequency of consumption of each category (daily, weekly, monthly, or a few times a year). In Q4, participants were asked to provide their primary motivation for consuming FFs as either taste, health benefits, cultural reasons, or other (free response). The following questions (Q5, Q6, Q7) asked participants to provide the sensory attributes, health benefits, and cultural relevance related to consuming FFs.

Fermented fruits and vegetables were the focus of the second and third block of questions in this survey. After providing background information on fermented fruits and vegetables, including delineating between pasteurized and fresh (refrigerated and non-pasteurized), participants were asked in Q8 if they consumed fermented fruits and vegetables. Participants were asked if the fermented fruits and vegetables they consumed required refrigeration in Q9. The next series of questions (Q10-Q14), focused solely on fresh fermented fruits and vegetables. Q10 asked participants to indicate the frequency of consumption of fresh fermented fruits and vegetables as either daily, weekly, monthly, a few times a year, or never. The same questions were asked for motivations for consumption as above for FFs in general including, the primary motivation for consuming fresh fermented fruits and vegetables, flavors associated with these foods, perceived health benefits associated with consumption, and cultural reasons for consuming fermented fruits and vegetables (Q11–Q14).

The final block of questions (Q15–18) focused on the in-home production of fermented fruits and vegetables and these data are not included in this analysis. These data are presented elsewhere because they are specific for Extension and food safety specialists (Hanlon et al., 2026). Demographic questions were also included in the survey (Q19–26) and asked participants to self-identify gender, age, ethnicity, race, level of education, country of residence, state of residence, and annual family income.

The developed questionnaire went through multiple internal reviews for clarity and ability to deliver desired research outcomes. The survey research questions, Qualtrics survey, and survey logic were validated by four external reviewers, one fruit and vegetable fermentation expert (government affiliated), two food safety extension specialists (university affiliated), and one evaluation specialist (university affiliated). Based on the feedback from external review, the survey tool was updated and then piloted with a group of 30 University of California Agriculture and Natural Resources (UC ANR) Master Food Preserver (MFP) volunteers. The pilot participants were asked to provide feedback on question clarity, functionality in Qualtrics, and estimated duration to completion. The survey was reviewed by the UC Davis Institutional Review Board (IRB) Administration (Project Title: [1657403–1] Survey of consumer consumption habits) and was deemed Exempt. All survey participants provided consent to complete the survey.

### 2.2 Data collection

The developed Qualtrics survey was open from April 2022 to September 2022. The survey was shared through multiple mechanisms. First, the survey was distributed to a listserv of approximately 500 contacts curated through outreach activities associated with the project team. The survey was shared via social media associated with the project team (Instagram, Facebook) and through home preservation and food safety extension networks. Finally, the survey was included in The Fermentation Association (TFA) newsletters. A total of 853 responses were collected, with 851 respondents consenting to participate in the survey.

### 2.3 Data processing and analysis

Participants with missing data were removed, for a total of 751 responses included in analysis. Missing data was defined as missing responses for Q19 through Q24. Entries with identical responses for both closed-end questions and free responses were also deleted. Frequency data was used for categorical variables and descriptive statistics. Open-ended questions were included intermittently in the survey to ensure closed questions were being properly addressed by respondents and to inquire for additional information when necessary (Singer and Couper, 2017). Income values were reported as an open response, so values were changed to numeric manually and any value range listed was changed to the respective average. Open-ended questions were analyzed via methods by previewing responses, subsequently developing a coding frame to encompass responses, and then manually assigning codes to each comment manually (O’Cathain and Thomas, 2004).

To examine correlations between questions, responses to certain items were grouped by unique categories and compared to the total number of respondents to a related question. Proportions were then calculated for each category, following the method used by Šikić-Pogačar et al. (2022). This method was used to draw comparisons between Q3 and Q6, Q4 and Q11, Q4 and Q22, Q1 and Q22, Q1 and Q20, Q1 and Q23, and Q1 and Q21. A two-way analysis of variance (ANOVA) with interaction effect was used to analyze differences in responses between Q3 and Q6 (King and Eckersley, 2019). A paired t-test was used to compare the mean responses for each matched question between the question block about non-alcoholic FFs (Q4, Q6, Q7) and the block related to fresh fermented fruits and vegetables (Q11, Q13, Q14) (Alasuutari et al., 2008). A *p*-value below 0.05 was considered significant. Data processing and analysis was performed using R studio and Python in Jupyter Notebook (Kluyver et al., 2016; R Core Team, 2022).

### 2.4 Limitations

As with all survey tools, there are inherent limitations to this study. While the participant pool was sufficiently large, the demographic data indicates a high proportion of respondents were female, aged 18–39, white, and had achieved a level of education above a high school diploma. The distribution methods for the survey, including university affiliated emails and social media posts, likely influenced this skewing of the sample population to a younger and educated population. Moreover, previous studies using non-targeted survey tools have found that individuals assigned female at birth are generally more likely to participate in surveys (Becker, 2022). Also, women are also the majority gender demographic currently enrolled in higher education (Parker, 2021). Although the survey was accessible to individuals outside the U. S. and advertised internationally, 87% (*n* = 656) of respondents resided in the United States. The remaining participants were primarily from Canada, Argentina, Brazil, and the United Kingdom. While the sample size is of sufficient power for analysis, correlations should not necessarily be extrapolated to demographic populations with low representation in the study. Lastly, although we attempted to disseminate the survey to a broad audience, because it was shared through listserv and social media accounts with audiences interested in fermented foods, the respondents may have more awareness of fermented foods than the general population.

## 3 Results

### 3.1 Demographics of survey respondents

A total of 751 participants were included in analysis after incomplete and duplicate entries were removed. As shown in Table 1, most respondents identified as female (*n* = 463; 61.65%) and the largest age group represented was 18–29 years (*n* = 302; 40.21%). Participants were also asked to report their highest level of education, with the majority reporting some level of higher education (*n* = 662; 88.14%). Most respondents identified themselves as White (*n* = 532; 70.84%) and resided within the United States (*n* = 656; 87.35%).

**Table 1:** Demographic responses of study participants.

Characteristics	Frequency	Percentage (%)
Gender		
Male	258	34.35
Female	463	61.65
TransMale	2	0.27
TransFemale	1	0.13
Non-binary	14	1.86
Prefer not to respond	13	1.73
Age		
18–29	302	40.21
30–39	232	30.89
40–49	106	14.11
50–59	49	6.52
60–69	45	5.99
70 and above	17	2.26
Ethnicity		
Hispanic or Latino(a)	158	21.04
Not Hispanic or Latino(a)	593	78.96
Race		
American Indian/Alaska Native	7	0.93
Asian	95	12.65
Black/African American	21	2.80
Native Hawaiian/ Other Pacific Islander	15	2.00
White	532	70.84
Two or more races	35	4.66
Other/not listed	18	2.40
Prefer not to respond	26	3.46
Highest education level		
No formal degree	19	2.53
High school degree or diploma	60	7.99
Associate’s degree	90	11.98
Bachelor’s degree	311	41.41
Master’s degree	185	24.63
Doctoral or professional degree	76	10.12
Prefer not to respond	9	1.20
Country of residence		
United States	656	87.35
Outside of United States	85	11.32
Prefer not to respond	10	1.33

Total response *n* = 751.

### 3.2 Participant identification of fermented foods

**Figure 1:**
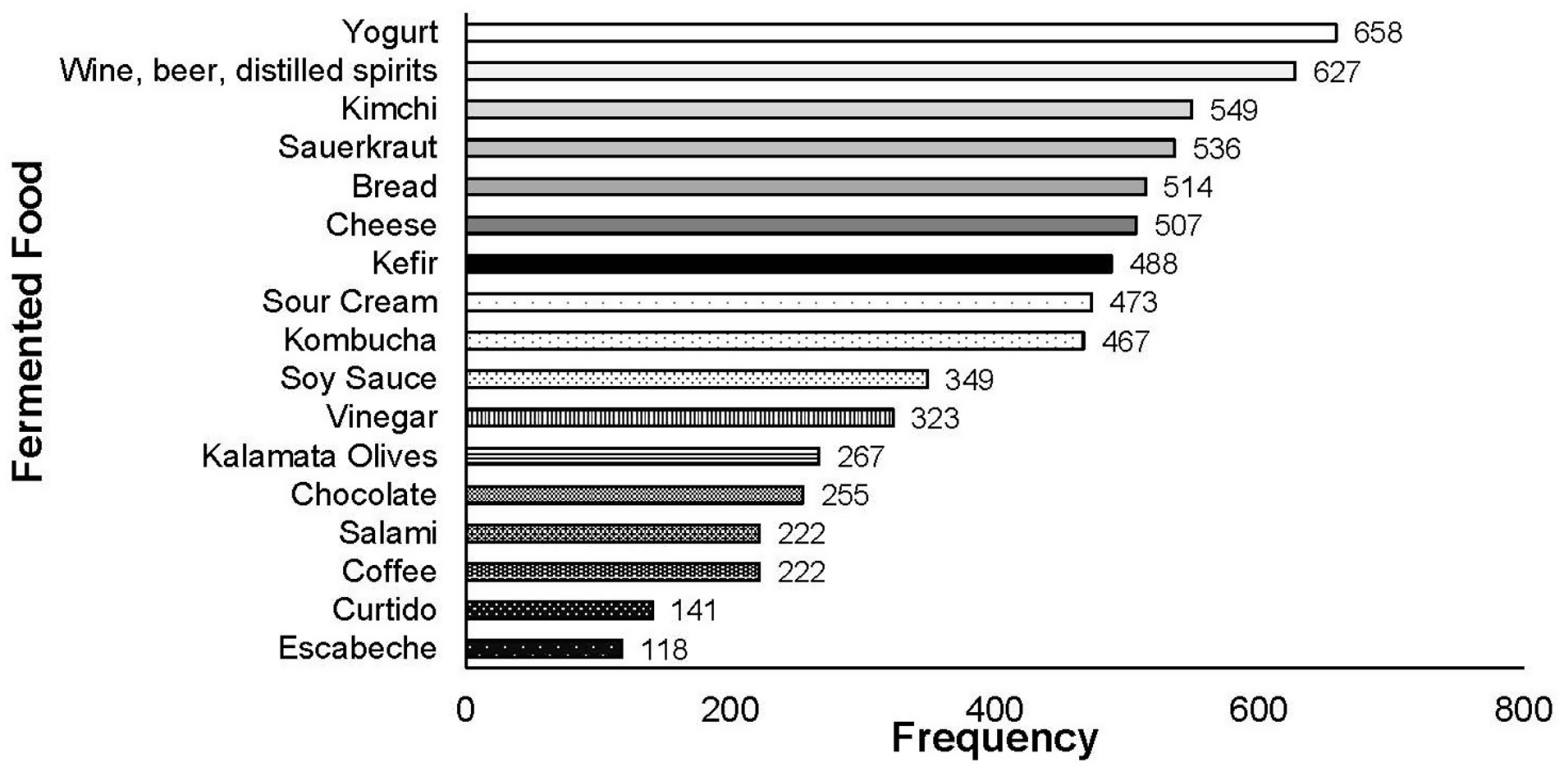
Response frequency for question 1: “Based on this explanation and your experience, please indicate below which of the following foods can be made by fermentation or are examples of FFs and beverages. Please check all response options that apply.” Numbers listed along *y* axis are total response frequency for that FF (*n* = 751).

Prior to the first question in the survey, participants were provided the definition of fermented foods [“foods and beverages made through desired microbial growth and enzymatic conversions of food components (Marco et al., 2021)]” (Supplementary Data, Supplementary Table 6). The first block of questions focused on the general category of FFs. In Q1, a list of fermented products was provided (yogurt, sour cream, kefir, cheese, wine, beer, distilled spirits, kalamata olives, bread, coffee, chocolate, kombucha, salami, vinegar, kimchi, sauerkraut, curtido, escabeche, soy sauce, or none of these) and participants were asked to identify which foods were produced through fermentation (Figure 1). All foods provided on the list were fermented, but only 7% (*n* = 55 of total 751) of respondents correctly identified all foods as fermented. Yogurt was the most frequently identified FF by participants (*n* = 658 of total 751; 87.62%). Alcoholic beverages (wine, beer, distilled spirits) were the second most identified type of FFs (*n* = 627 of total 751; 83.49%). Kimchi (*n* = 549 of total 751; 73.10%) and sauerkraut (*n* = 536 of total 751; 71.37%) were the third and fourth most frequently identified FF. A high proportion of participants also correctly identified other fermented dairy products, such as cheese (*n* = 507 of total 751; 67.51%), kefir (*n* = 488 of total 751; 64.98%), and sour cream (*n* = 473 of total 751; 62.98%). Kombucha was also identified as an FF by over half of survey participants (*n* = 467 of total 751; 62.18%). Other FFs were correctly identified by less than half of survey participants. Curtido (*n* = 141 of total 751; 18.77%) and escabeche (*n* = 118 of total 751; 15.71%) were least frequently recognized.

### 3.3 Participant reported consumption of different categories of non-alcoholic fermented foods

**Figure 2:**
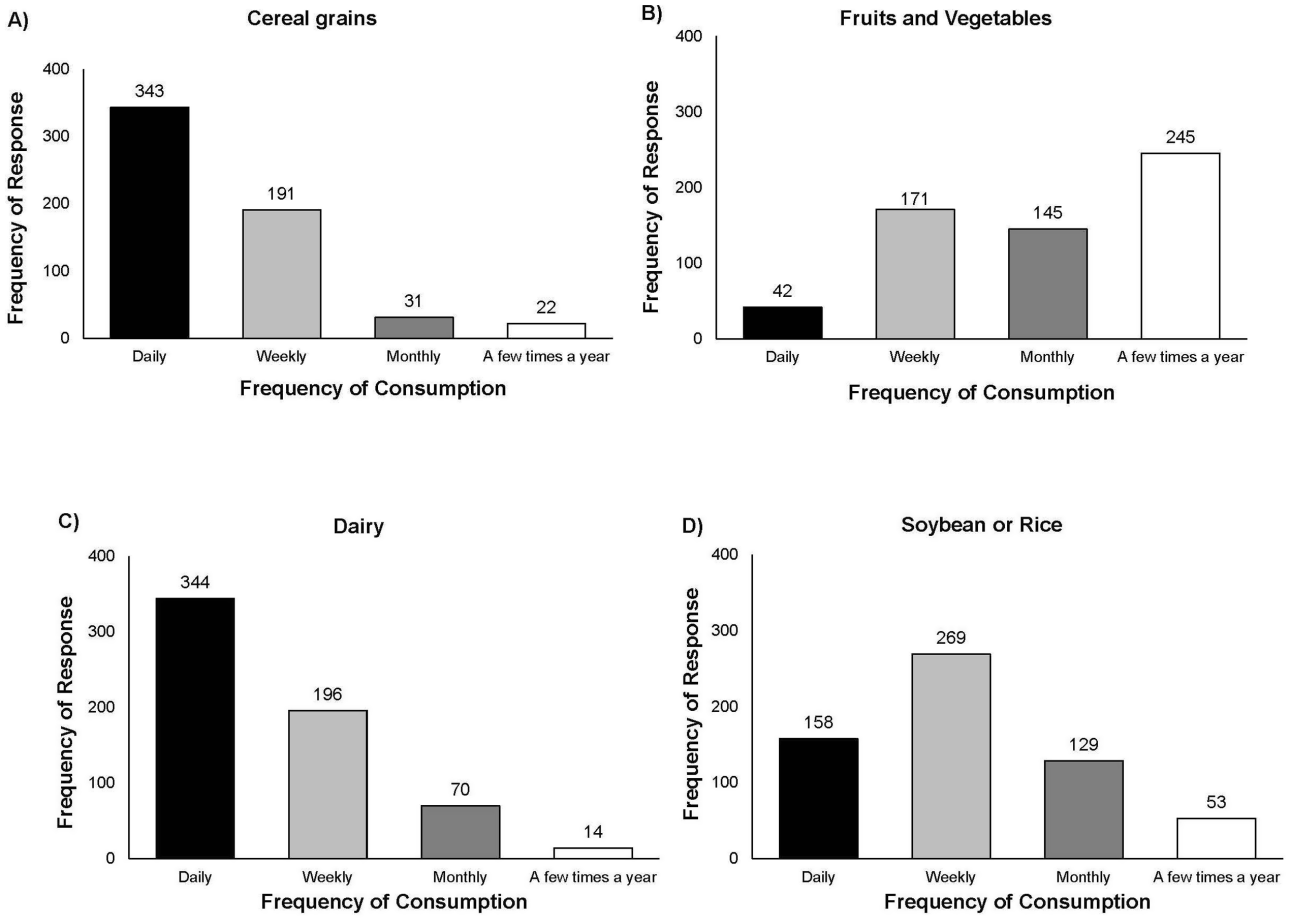
Responses for Q3: “Which types of FFs do you eat (excluding alcoholic beverages)? Please indicate the frequency of consuming that food by checking the box next to: daily, weekly, monthly, or a few times a year.” Total responses for Q3 *n* = 665. Fermented cereal grains (*n* = 307; 46.17%) was the category of FFs with the highest reported frequency of consumption with over half or respondents reporting consuming these foods daily (*n* = 343; 51.58%). A total of 42.26% (*n* = 281) participants indicated consuming fermented fruits and vegetables, 39.70% (*n* = 264) consumed fermented dairy products, 30.68% (*n* = 204) reported consuming fermented meats, and 37.60% (*n* = 250) reported consuming fermented rice or soybean products.

Participants were asked in Q2 if they consumed non-alcoholic FFs and beverages, (excluding beer, wine, and distilled spirits) to which 85.22% (*n* = 640 of total 751) responded “Yes” (Supplementary Data, Supplementary Table 7). Participants were asked to indicate which categories of non-alcoholic FFs and beverages (cereal grains, fruits and vegetables, dairy, soybean or rice, meats, and other) they consumed and to indicate the frequency of consumption of each category as daily, weekly, monthly, or a few times a year (Figure 2, Supplementary Data, Supplementary Table 8). Participants indicated consuming fermented fruits and vegetables and fermented soybean or rice products most frequently on a weekly basis, as compared to daily for fermented dairy, and monthly for meats.

### 3.4 Motivators for consuming non-alcoholic fermented foods including sensory attributes, health benefits, and cultural reasons

**Figure 3:**
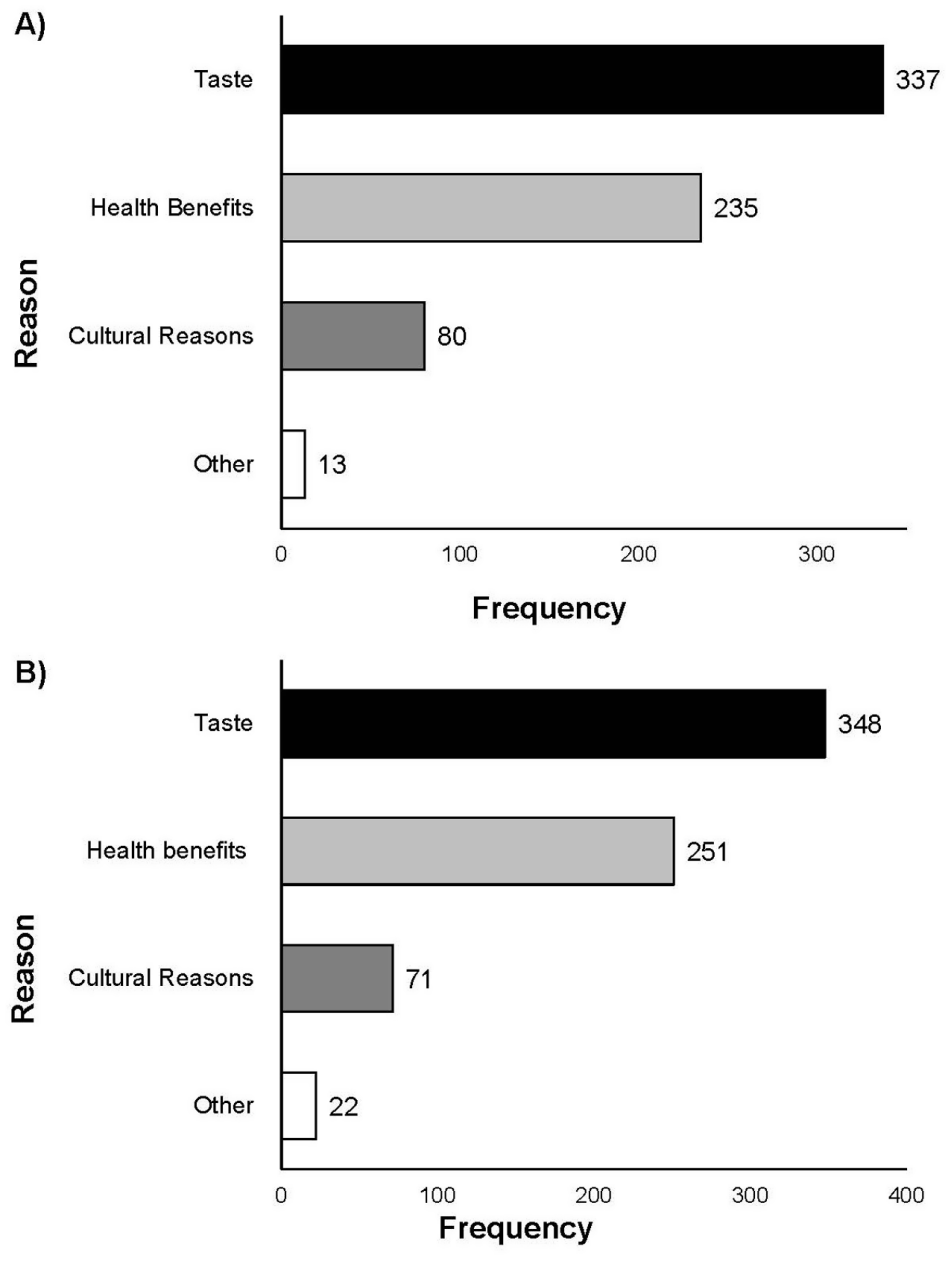
Responses for Q4 **(A)** and Q11 **(B)**: “what is your main reason for eating [non-alcoholic FFs]/[fresh fermented fruits or vegetables]?” Single response allowed. Total response for Q4 (*n* = 665) and for Q11 (*n* = 694).

Participants were asked to select one answer for the primary motivator for consuming FFs as either (1) taste, (2) health benefits, (3) cultural reasons, or (4) other, in Q4. Most respondents indicated that “taste” was the primary reason for consuming FFs (*n* = 337 of total 665; 50.68%) (Figure 3A). “Health benefits” was the second highest frequency response (*n* = 235 of total 665; 35.34%), followed by “cultural reasons” (*n* = 80, of total 665 12.03%). A total of 1.95% (*n* = 13 of 665) respondents selected “other” with responses including “enjoying the fermentation process” and “food waste prevention”. Regardless of the primary motivator selected in Q4, all participants were asked to complete Q5, Q6, and Q7 related to sensory attributes, health benefits, and cultural motivators related to or associated with the consumption of non-alcoholic FFs.

**Figure 4:**
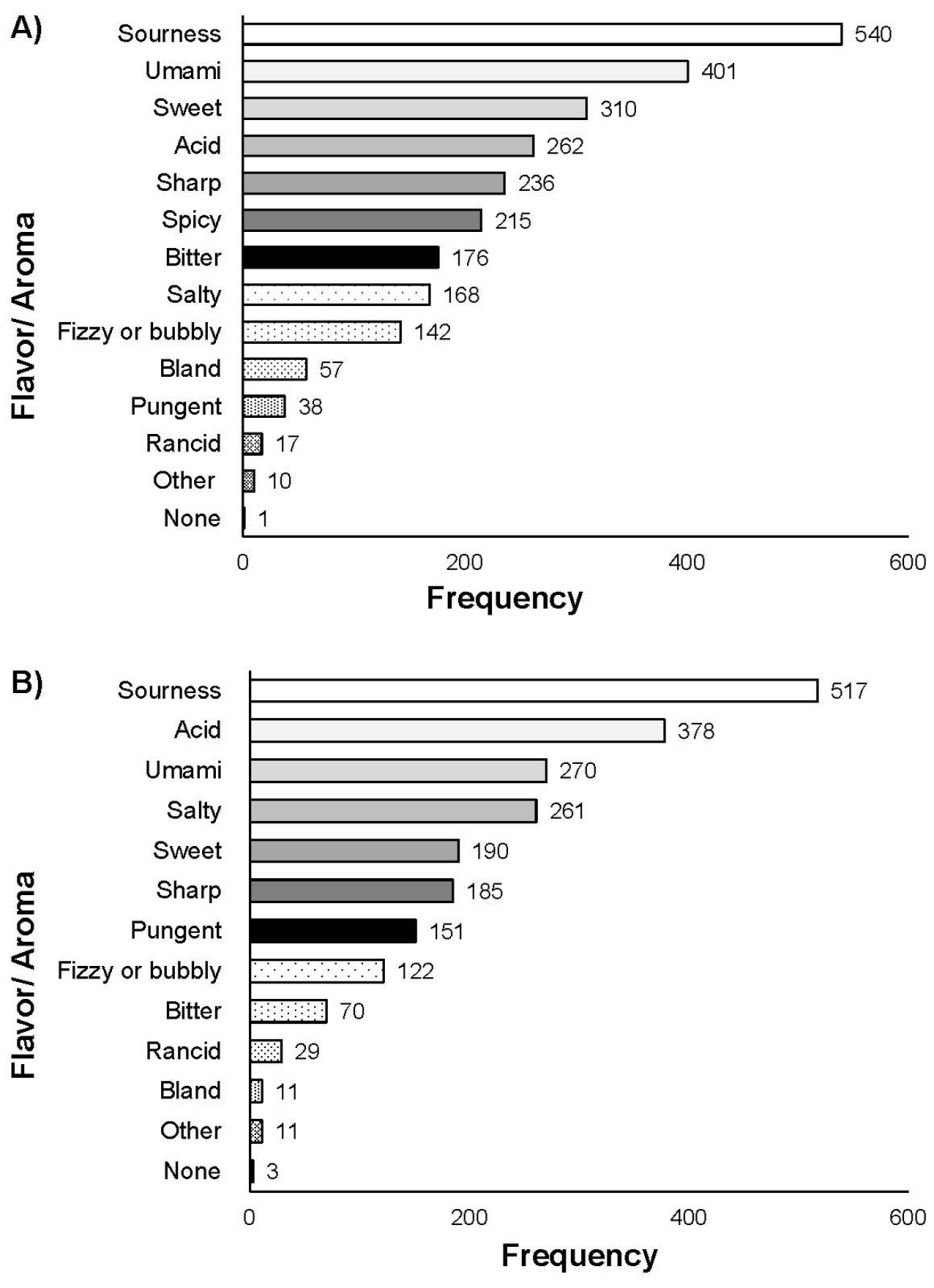
Responses for Q5 **(A)** and Q12 **(B)**: “What flavors or aromas do you associate with [FFs]/[fermented fruits or vegetables]? Select all that apply.” Total responses for Q5 (*n* = 665) and for Q12 (*n* = 694).

In Q5, participants were asked to select all the flavors and/or aromas associated with FFs from a list of 12 (sourness, umami, sweet, acid, sharp, spicy, bitter, salty, fizzy or bubbly, bland, pungent, rancid, other, none). Sourness (*n* = 540 of total 665; 81.20%) and acid (*n* = 401 of total 665; 60.30%) were the most frequent responses, while bland (*n* = 17 of total 665; 2.56%) and rancid (*n* = 38; 5.71%) were least frequently chosen (Figure 4A). Participants selecting “other” (*n* = 10 of total 665; 1.50%) were asked to provide a write in text answer to which responses included: yeasty, fruity, tangy, or earthy.

**Figure 5:**
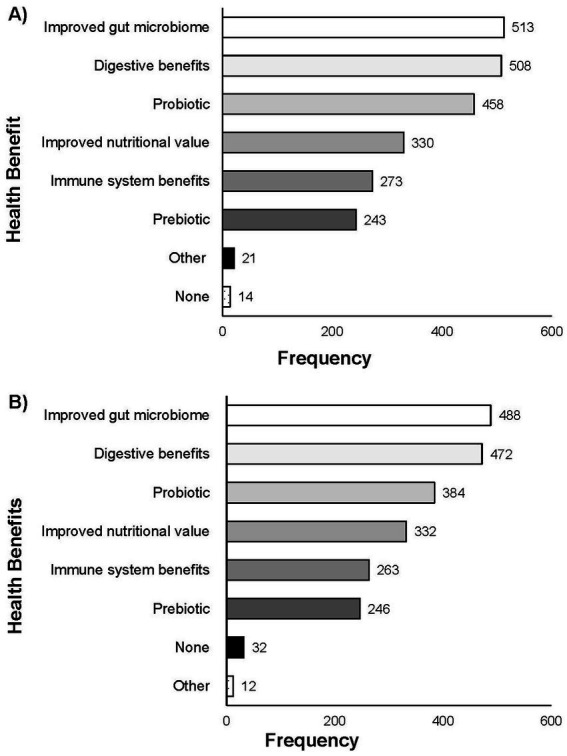
Responses for Q6 **(A)** and Q13 **(B)**: “What, if any, health benefits do you associate with the consumption of [FFs]/[fresh fermented fruits and vegetables]? Select all that apply.” Total responses for Q6 (*n* = 665) and for Q13 (*n* = 694).

In Q6, participants were asked to indicate which (if any) health benefits they associated with the consumption of FFs (digestive benefits, improved gut microbiome, probiotic, prebiotic, improved nutritional value, immune system benefits, other, none). The most highly selected health benefits associated with FF consumption were “improved gut microbiome” (*n* = 513 of total 665; 77.14%), “digestive benefits” (*n* = 508 of total 665; 76.39%), and “probiotic” (*n* = 458 of total 665; 68.87%) (Figure 5A). Participants also indicated associating “improved nutritional value” (*n* = 330 of total 665; 49.62%), “immune system benefits” (*n* = 273 of total 665; 41.05%), and “prebiotic” (*n* = 243 of total 665; 36.54%) with the consumption of FFs.

FF consumption frequency data (Q3) for participants that selected “digestive benefits,” “improved gut microbiome,” or “probiotic” as the primary health benefits associated with FF (Q6) were compared (Supplementary Data, Supplementary Table 25). Results of a two-way ANOVA with interaction effect for the reason (i.e., probiotic) and category (i.e., dairy) against response frequency found no significant differences (*p* = 0.73). For example, responses of individuals that selected probiotic as a health benefit was not significantly different when comparing between fermented dairy and fermented fruits and vegetables (*p* = 0.2). Thus, health benefits were not associated with a specific category of fermented foods, but the health benefit attribute was associated with all fermented foods.

**Figure 6:**
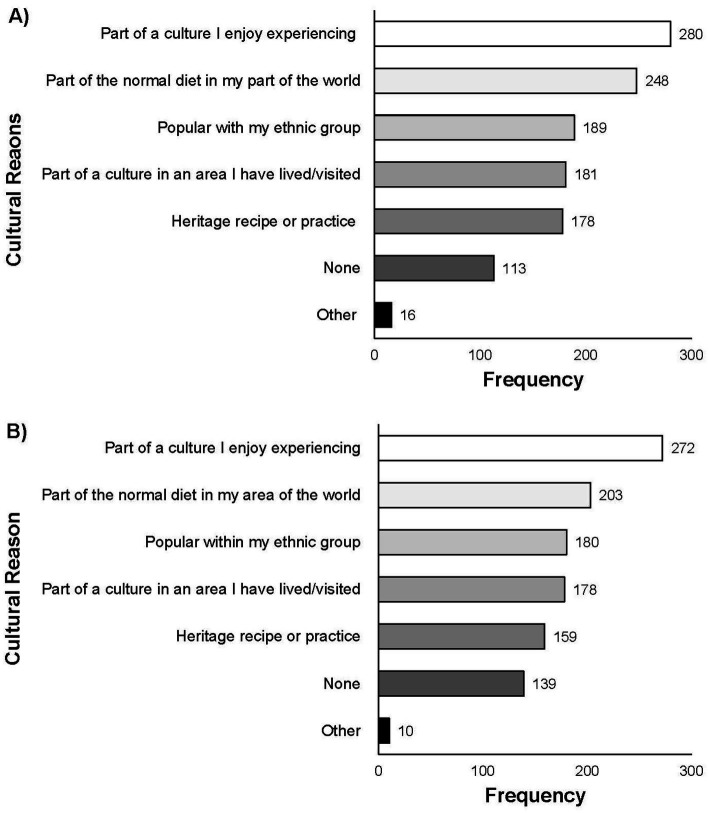
Responses for Q7 **(A)** and Q14 **(B)**: Are there any cultural reasons you consume [non-alcoholic FFs and beverages]/[fresh fermented fruits and vegetables]? Select all that apply.” Total responses for Q6 (*n* = 665) and for Q13 (*n* = 694).

Participants were then asked to provide any cultural reasons for consuming non-alcoholic FFs in Q7. The most selected cultural reason for consuming FFs was “part of a culture I enjoy experiencing” (*n* = 280 of total 665; 42.11%) (Figure 6A). Participants also selected “part of the normal diet in my part of the world” (*n* = 248 of total 665; 37.29%), “part of a culture in an area I have lived or visited” (*n* = 181 of total 665; 27.22%), “popular with my ethnic group” (*n* = 189 of total 665; 28.42%), and “heritage recipe or practice” (*n* = 178 of total 665; 26.77%). If participants selected “other” (*n* = 16 of total 665; 2.41%) they were asked to provide a free response to which responses included “part of a community affiliation” or having a career as a “chef” or “nutritionist.”

### 3.5 Consumer purchasing habits of fermented fruits and vegetables

The next question (Q8) addressed whether consumers were purchasing fermented fruits or vegetables. A total of 398 respondents out of 751 (53.00%) indicated they purchased fermented fruits and vegetables. Of those responding yes, some provided the type of fermented fruits and vegetables they purchased with coded free responses including “kimchi,” “sauerkraut,” “olives,” “hot sauce,” “umeboshi plums,” “citrus,” and “pickles.” Others listed non-fruit or vegetable fermented products including “yogurt,” “natto,” and “sourdough.” Respondents were asked if they purchase fresh fermented fruits and vegetables that require refrigeration to which 58.87% (*n* = 282 of total 751) responded yes.

### 3.6 Consumption frequency and motivators related to fresh fermented fruits and vegetables

In Q10 participants were provided background information and a definition of fresh fermented fruits and vegetables, “Fresh fermented fruits and vegetables are consumed directly after fermentation or after storage in the refrigerator. Fresh fermented fruits and vegetables have not gone through a process to make them shelf stable (thermal processing). You can find a fresh fermented fruit or vegetable in the refrigerated section of the grocery store or sold chilled at Farmers’ Markets.” and then asked how often they consumed these foods as either daily, weekly, monthly, a few times a year, or never. Of those that did consume fresh fermented fruits and vegetables 21.76% (*n* = 151 of total 751) ate the products daily, 36.74% (*n* = 255 of total 751) ate the products weekly, 23.20% (*n* = 161 of total 751) ate the products monthly, and 18.01% (*n* = 125 of total 751) consumed these products a few times a year. The participants were asked to select one primary motivator for consuming fresh fermented fruits and vegetables (Q11), 50.14% (*n* = 348 of total 694), selected taste, 36.17% (*n* = 251 of total 694) reported health benefits, 10.23% (*n* = 71 of total 694) indicated cultural reasons (Figure 3B). Responses from those that selected “other” included “reduced cost,” “food preservation,” and “enjoying fermentation.” Comparisons between all FF and fresh fermented fruits and vegetables with regard to main reason for consumption revealed that there was no significant difference between the responses (*p* = 0.92).

In Q12, participants were asked what flavors and/or aromas they associate with fresh fermented fruits or vegetables. Sourness (*n* = 517 of total 694; 74.50%) and acid (*n* = 378 of total 694; 54.47%) were most frequently selected (Figure 4B). Open responses to “other” included “spicy,” “fruity,” “tangy,” “earthy,” and “crunchy.” In Q13, participants were asked what health benefits they associate with the consumption of fresh fermented fruits and vegetables. Health benefits most associated with fresh fermented fruits and vegetables was “improved gut microbiome” (*n* = 488 of total 694; 70.32%), “digestive benefits” (*n* = 472 of total 694; 68.01%), “probiotic” (*n* = 384 of total 694; 55.33%), and “improved nutritional value” (*n* = 332 of total 694; 47.84%) (Figure 5B). When comparing the associated health benefits for all fermented food to specifically fermented fruits and vegetables (comparison of responses to Q6 and Q13) there was no significant difference in the response was found (*p* = 0.05).

In Q14, participants were asked if there are cultural reasons for which they consume fermented fruits or vegetables. The most selected cultural reasons were “part of a culture I enjoy experiencing” (*n* = 272; 39.19%), “part of the normal diet in my area of the world” (*n* = 203 of total 694; 29.25%), and “popular within my ethnic group” (*n* = 180 of total 694; 25.94%) (Figure 6B). When comparing the cultural reasons for consuming all fermented food to specifically fermented fruits and vegetables (comparison of responses to Q7 and Q14) there was no significant difference in the responses (p = 0.05).

### 3.7 Comparisons of demographics with survey responses

**Figure 7:**
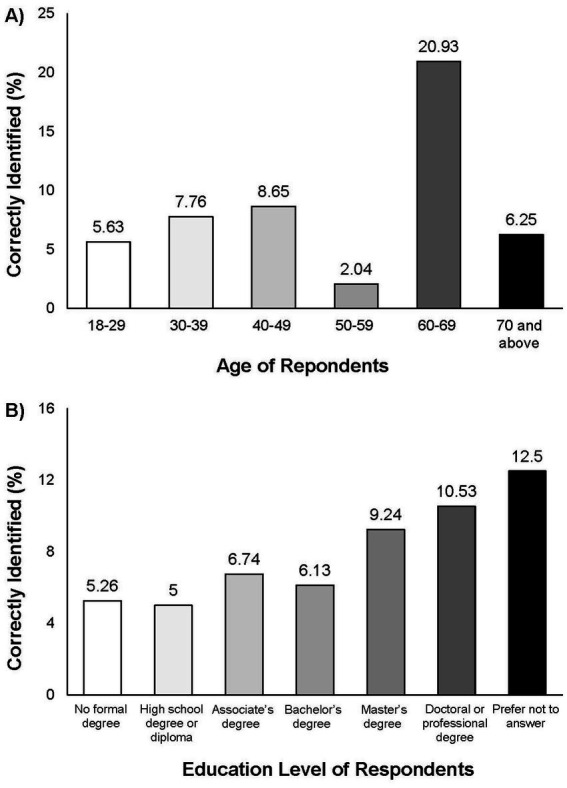
Percentages of total responses to Q20 **(A)** and Q23 **(B)** calculated from respondents that selected all foods as fermented in Q1 (*n* = 55).

We compared demographic data to correct identification of all FFs listed in Q1. There were correlations identified associated with age and education status of participants. Of all age groups, the largest percentage of respondents that correctly identified all foods as fermented were between 60 to 69 (*n* = 9 of total 45; 20.93%) (Figure 7A). Of all reported education levels, the largest proportion of respondents to correctly identify all FFs correctly were participants with a doctoral or professional degree (*n* = 8 of total 76; 10.53%) (Figure 7B).

Curtido and escabeche are traditionally Hispanic fermented products, so we identified which participants selected curtido and escabeche, not exclusively, for Q1 and compared with responses for ethnicity, Q21 (Supplementary Data, Supplementary Table 22). In total, 14.01% (*n* = 22 out of total 158) of Hispanic or Latino(a) participants identified escabeche as fermented while 19.11% (*n* = 30 out of total 158) of Hispanic or Latino(a) participants identified curtido as fermented. Of not Hispanic or Latino participants, 16.3% (*n* = 96 out of total 593) identified escabeche as fermented and 18.85% (*n* = 111 out of total 593) identified curtido as fermented.

Similarly, kimchi is a traditional Asian FF, so we identified which participants selected kimchi in Q1 and compared with responses for race, Q22. Participants that identified as being two or more races had the largest proportion to correctly identify kimchi (*n* = 31 of total 35; 88.57%) (Supplementary Data, Supplementary Table 23). In total, 86.17% (*n* = 81 of total 95) of participants that identified themselves as Asian correctly identified kimchi as fermented. The lowest proportion of participants that selected kimchi as fermented identify as Native Hawaiian/ Other Pacific Islander (*n* = 8 of total 15; 53.33%).

## 4 Discussion

Fermented foods are dietary staples, but knowledge on and motivations for consumption of fermented foods among the general populous in the US is not well known. This study examined consumer awareness of the variety of foods that are produced via fermentation, reasons why they choose fermented foods, and some common perceptions about FFs. Overall, participants in the survey were familiar with many categories of foods that can be processed via fermentation, including dairy, grains, alcoholic beverages, non-alcoholic beverages and fruits and vegetables. Consumption frequencies of non-alcoholic FFs varied by category, with fermented grain (bread) and dairy (yogurt) being consumed on the most frequent basis. Participants were primarily drawn to non-alcoholic FFs for taste, however they also identified several health promoting benefits they associated with the consumption of FFs.

Although participants were familiar with many FFs included in this survey, there were several types of foods which were less frequently identified. For example, two cabbage-based fermentations (kimchi and sauerkraut) were identified by most participants while curtido (another cabbage-based fermentation) was identified by less than one fourth of participants as fermented. These are all fermented cabbage products, however the data indicate that there is bias toward familiarity of FFs associated with the cuisine of European or Asian countries among respondents. Curtido is made from cabbage, onions, and carrots and is traced back to El Salvador (Chilton et al., 2015). There was no correlation between respondent’s ethnicity (Hispanic/Latino or Not Hispanic/Latino) and correct identification of curtido as an FF in this study, it is likely the ethnic origin of this traditional FF led to it being less recognizable to study participants.

Other less frequently identified FFs by participants included soy sauce, Kalamata olives, coffee and chocolate. Soy sauce is traditionally fermented but is also produced commercially via hydrolysis of soybeans using high heat and acid (Liu, 2008; Hutkins, 2018; Sassi et al., 2021). The resulting “formulated soy sauce” is the common type of soy sauce encountered in most U. S. supermarket chains (Farnworth, 2003). Table olives can also be prepared using non-fermentation-based methods such as lye curing or dry salt curing (Yada et al., 2007). It is possible that individuals are more familiar with the chemical processing of these two foods, which may have led to decreased recognition of these as FFs. Similarly, coffee beans are not always fermented and this may have led to the decreased level of identification of coffee as a fermented food. The fermentation process of both chocolate and coffee occurs at the country of origin immediately after harvesting (Cortés et al., 2024; Campos et al., 2025). Most U. S. residents, which comprise the majority of this sample pool, would not encounter cocoa or coffee fermentation as this activity is limited to select portions of the globe. The FF products encountered by many respondents would likely be products processed post fermentation, and that may limit awareness of both chocolate and coffee.

As found previously (García-Barón et al., 2025), taste was identified as the primary motivator for consumption within survey participants. Sensory characteristics such as ‘sourness’ and ‘acid’ were the most frequently associated organoleptic properties of non-alcoholic FFs among participants. Sour and acid tastes are imparted by the production of organic acids during fermentation (Johanningsmeiner et al., 2005). Many of the FF types frequently identified by participants (yogurt, cheese, sour cream, kimchi, sauerkraut) are fermented by lactic acid bacteria (LAB), which produce lactic acid as one by product of fermentation (Wei et al., 2025). It is possible that the association of the flavor profile of these highly recognized FFs by participants resulted in the major association of these flavors with all FFs. By comparison, the umami flavor in FFs is attributed to the presence of glutamate above 1 mM and is typically found in fermented cheese, soy sauce, meat, bread, or fish (Zhao et al., 2016). The lower frequency of responses identifying umami as a flavor compared to sourness may be attributed to a lack of familiarity with the flavor or the types of FFs typically associated with umami.

The most frequently identified health benefits participants associated with fermented food consumption included improved gut microbiome, digestive benefits, and probiotic. While participants identify improved gut microbiome as a key health benefit, they may assume that live microorganisms consumed in fermented foods incorporate into their own gut microbiome. This is evidenced by the low response rate for prebiotic and improved nutritional value, which are supported by the literature as being components of FFs that support the gut microbiome (Marco et al., 2021). Prebiotics are “substrate[s] that [are] selectively utilized by host microorganisms conferring a health benefit” and these compounds can be present in FFs as initial ingredients or through in-situ synthesis from microorganisms (Gibson et al., 2017; Marco et al., 2021). A study among Romanian consumers in 2018 found that while 74% of respondents were familiar with the term prebiotics, the definition was not well understood (Precup et al., 2022). It is known that prebiotics and generation or conversion of other molecules increases the nutritional value of FFs, increased education around these benefits is required for consumers as well as FF manufacturers (Christensen et al., 1999; Moore et al., 2022).

Over two-thirds of respondents reported associating probiotic health benefits with non-alcoholic FFs and beverages. This finding may be due to the general notion that FF may contain live microbes. Whereas probiotics are defined at the strain level and must be shown to confer a health benefit in randomized controlled trials, the microorganisms in FF are generally undefined, present at unknown levels, and not shown to confer a health benefit (Marco et al., 2021). Probiotic fermented dairy products were one of the first probiotic foods available to consumers (Yilmaz-Ersan et al., 2020). Consumer focus groups conducted in California in 1998, found variations in consumer awareness and perception of probiotics in dairy products, ranging from unaware to having some level of understanding of the health benefits associated with probiotic consumption (Bruhn et al., 2002). A study conducted in Brazil in 2007 found that 29.05 and 21.67% of individuals interviewed were able to correctly define and identify a probiotic food, respectively (Viana et al., 2008). A 2018 study conducted in Turkey found 43.95% of participants were familiar with probiotic dairy products, while 56.05% had never heard of probiotic dairy products (Yilmaz-Ersan et al., 2020). Beyond probiotic dairy, today consumers may also encounter probiotic juices, sodas, cereal based bars, candies, teas, among others (Dey, 2018). While consumers may be more familiar with probiotic products today, it is unclear how and if they understand the distinction of probiotics and FFs. Thus, it would be informative to explain that not all FF contain live microbes and only certain FF have added probiotic strains. Such strains should be defined on the product label with the numbers of the probiotic strain present until the end of the product shelf life.

Although these findings provide new perspectives on how the public understands fermented foods, the survey and its findings have several limitations. Firstly, the question related to motivations for FF consumption only allowed participants to select one response, which may be a limitation as food choice decisions are often very complex and involve multiple motivators. Participants still had an opportunity to provide responses to the other motivating factors listed in the question in the subsequent questions in the survey and data related to these factors in motivating fermented food choices are explored. Secondly, the terms used to describe sensory attributes were based on sensory studies and the terminology used in those publications to describe characteristics of fermented foods. It is possible that some of the terminology was not familiar to untrained participants and that may limit the interpretation of results presented here. Additional studies utilizing a properly trained panel of participants could more accurately determine consumer preferences related to flavor and aroma. Moreover, in the survey tool, a definition of FFs, pasteurized (shelf stable) FFs, and non-pasteurized (fresh) FFs were provided to participants (Supplementary Materials, Supplementary Tables 1–3). Definitions of probiotic and prebiotic were not provided to study participants, which is a study limitation. Moreover, to minimize survey fatigue, the probiotics and prebiotics terms were included within the general category of health benefits. However, because those terms intend to convey functional components of food and not directly a health benefit, that placement may have led to confusion among participants. Previous work has shown both health professionals based in Europe and Australia and the public in Saudi Arabia have moderate knowledge of probiotic and prebiotics, and therefore it was an important concept to gage among participants in this study (Fijan et al., 2019; Alkhaldy, 2024). At the time the survey tool was developed (2021), the team relied on perceived health benefits associated with fermented foods based on websites and online news articles (American Heart Association, 2021; Harvard Health Publishing, Harvard Medical School, 2021; Today’s Dietitian, 2018) along with recommendations from our four external reviewers. Beyond the listed health benefits that could be selected, participants also had to option to select “other” and enter a text response for health benefits beyond those included as a multiple-choice option in the survey tool. If participants selected “other” (Supplementary Materials, Supplementary Tables 11, 12) text responses included “mood,” “mental health,” “sleep,” and “memory.” A more detailed future study should evaluate a broader subject base and expand on the health benefit component to make broader claims on consumer understanding of this motivating factor for fermented food consumption. This follow-up study could also explore fresh (non-pasteurized) FFs and probiotic foods to gain insight on consumer knowledge on these types of products.

Health related claims are increasingly present on food labels and often influence consumers preferences when selecting food items (Grasso et al., 2017; Ardeshiri and Rose, 2018; Grahl et al., 2018; Plasek and Temesi, 2019; Ballco and Gracia, 2020; Domínguez Domínguez Díaz et al., 2020; Benson et al., 2018; McSweeney, 2022). While sensory characteristics are still the primary driver of consumer food selection, extrinsic factors such as health-related claims can influence food choice (Marino et al., 2017; Crucean et al., 2019; McSweeney, 2022). In one study of fermented milk, a probiotic claim did not influence the overall participants liking of a product (Conti-Silva and De Souza-Borges, 2019; McSweeney, 2022;). In another study, fruit juice acceptability increased when probiotic claims were included for a product (Pereira et al., 2017; McSweeney, 2022). Only those products containing probiotic cultures at appropriate dosages should be labeled as probiotic, whereas fresh fermented foods could bear a statement “contains live and active cultures” (McSweeney, 2022). Proper labeling of these products is imperative to reduce misleading consumers, but also to ensure the health promoting benefits of consuming FFs is adequately conferred to individuals seeking healthy diets. It has been hypothesized that health-related claims may also help with acceptance of unfamiliar foods (McSweeney, 2022). Therefore, labeling fresh fermented foods with “contains live and active cultures” could be a driver to motivate consumers to try new varieties of FFs.

Participants identified digestive benefits as a health benefit conferred by the consumption of FFs. A systematic review of human trials conducted on health impacts associated with the consumption of fermented dairy show evidence of favorable effects on gastrointestinal (GI) health associated with consumption in 20 of 26 total studies, with no significant impact in the remaining studies (Savaiano and Hutkins, 2021). Another narrative review evaluating human observational and randomized control trials (RCTs) investigating impacts of consumption of fermented dairy products on GI symptoms and/or GI biomarkers of health found that of 37 studies, 10 reported no benefit on GI health while the remainder reported some associated GI health benefit (Bui and Marco, 2025). While human trials generally support modest but consistent GI health benefits associated with consumption of fermented dairy products, several studies show no clear clinical benefit or very limited effects. For example, there was no difference in short chain fatty acid profiles or GI permeability in subjects consuming fermented dairy products as compared to placebo (acidified milk) (Zaccaria et al., 2023). Additionally, supplementation of fermented milk with probiotic strains and/or dietary fiber showed no significant difference in the overall improvement of symptoms within subjects with irritable bowel syndrome compared to the placebo group (heat treated fermented milk without probiotics or fiber) (Šmid et al., 2016). An RCT has shown sauerkraut consumption to reduce irritable bowel syndrome symptoms, however this effect was observed for both pasteurized and non-pasteurized sauerkraut suggesting this impact was not conferred by consumption of live lactic acid bacteria (Nielsen et al., 2018). Moreover, bioactive compounds produced by metabolism of food components by the microorganisms involved in fermentation can also have beneficial impacts on gut health (Sonnenburg and Bäckhed, 2016; Mukherjee et al., 2024). Other types of health benefits have been reported in human trials conducted with fermented dairy including cardiovascular, weight, diabetes, and bone health (Savaiano and Hutkins, 2021) which were not included in the survey tool. The exclusion of these other potential health benefits may have biased participant data toward GI health benefits compared to other benefits of FF consumption. It remains challenging to interpret data to support health related claims for FFs due to the level of complicating factors for conducting such human trials and both the complexity and variety of FFs. Study participants here identify GI health benefits for FFs which are not currently supported by human trials and improved public facing communication regarding health benefits and FFs is needed to increase understanding among consumers.

Participants indicated that fermented foods are a “part of a culture that the participant enjoys experiencing” (42.11%) and that are a “normal part of the diet in the participants part of the world” (37.29%). A review of FF consumption in Africa from 1985 to 2008 found decreased consumption in urban areas compared to rural areas and this was attributed to increased westernization of diets in urban areas and loss of generational knowledge related to FF nutritional and health benefits (Anukam and Reid, 2009). The increased availability of fermented products such as yogurt, bread, sour cream, sauerkraut and kimchi, some of the most frequently identified non-alcoholic FFs by participants in this survey, may lead to a reduced diversity of lesser known, regional ferments (Materia et al., 2021). A study of lesser-known FFs in Eastern Europe found that knowledge of FF ingredients and processes has decreased, and thus more traditional fermentations have become marginalized (Sõukand et al., 2015). Small-scale fermenters offer the potential to invest in more artisanal FFs, with potential for greater diversity supporting a more heterogenous landscape of the industry (Flachs and Orkin, 2021).

Results from this survey suggest that while consumers have strong interest in fermented foods, there is lack of understanding of the diversity of FFs. Even with a provided definition of what qualifies a food as fermented, less than one tenth of participants correctly identified all FFs. These data highlight that more education is needed to help consumers understand FF from non-fermented products. Taste was identified as the primary reason for consumption by approximately 50% of participants. However, a single response was allowed for this question, and nearly 35% of participants selected health benefits as the primary reason for consuming fermented food. While some misconceptions exist about the type of health promoting benefits imparted by the consumption of FFs, these data give insight into an opportunity to leverage what is known about these benefits to enhance consumption of FFs in a wider population.

## Data Availability

The original contributions presented in the study are included in the article/Supplementary material, further inquiries can be directed to the corresponding author.
