# Supplementary material for: Consumer knowledge and motivations for consumption of fermented foods
Source: Front Microbiol. 2026 Apr 10;17:1789825. doi: 10.3389/fmicb.2026.1789825 (PMC13106139; doi:10.3389/fmicb.2026.1789825)
Supplement: Supplementary file 1 [file Table_1.docx]

Supplementary Material

# Supplementary Tables

**Supplementary Materials, Table 1.** Survey question block related to all FFs.

| **Question block 1: related to all fermented foods** | |
| --- | --- |
| Background information  (prior to Q1) | FFs and beverages are popular foods and could provide beneficial health effects. Making any type of FF requires a waiting step during preparation where the food or beverage is held for a period of time before getting the finished fermented product. Frequently that step is performed at room (or similar) or warmer temperatures (for example, letting dough rise). FFs and beverages are made through desired microbial growth and enzymatic conversions of food components. These foods and beverages are not the same as those that are preserved by adding acid such as vinegar, and the food is immediately canned or baked after making. |
| **Question (Q) number and type of response allowed** | **Question text included in survey** |
| Q1  (multiple selections allowed) | Based on this explanation and your experience, please indicate below which of the following foods can be made by fermentation or are examples of FFs and beverages. Please check all response options that apply.   - Yogurt - Sour cream - Kefir - Cheese - Wine, beer, distilled spirits - Kalamata olives - Bread - Coffee - Chocolate - Kombucha - Salami - Vinegar - Kimchi - Sauerkraut - Curtido - Escabeche - Soy sauce - None of these |
| Q2  (single response allowed) | Besides alcoholic beverages (example: beer, wine, spirits), there are many other types of FFs and beverages. Do you currently consume non-alcoholic FFs and beverages?   - Yes - No (if No, then skip to Q8) - Uncertain |
| Q3  (multiple responses allowed and free response for “other”) | Which types of FFs and beverages do you eat (excluding alcoholic beverages)? Please check the box next to the category of FFs and beverages you eat and indicate the frequency of consuming that food by checking box next to: daily, weekly, monthly, or a few times a year   - Cereal grain (such as bread) - Fruits and vegetables (such as sauerkraut, fermented pickles, kimchi) - Dairy (such as yogurt, cheese, kefir) - Soybean or rice (such as miso or soy sauce) - Meats (such as salami) - Other (please describe) |
| Q4  (single response allowed and free response for “other”) | What is your main reason for eating non-alcoholic FF and beverages?   - Taste - Health benefits - Cultural reasons - Other (please specify) |
| Q5  (multiple responses allowed and free response for “other”) | What flavors or aromas do you associate with FFs?  Select all that apply.   - Sourness - Umami - Sweet - Acid - Sharp - Spicy - Bitter - Salty - Fizzy or bubbly - Bland - Pungent - Rancid - Other (please specify) - None |
| Q6  (multiple responses allowed and free response for “other”) | What, if any, health benefits do you associate with non-alcoholic FFs and beverages?  Select all that apply.   - Digestive benefits - Improved gut microbiome - Probiotic - Prebiotic - Improved nutritional value - Immune system benefits - Other (please specify) - None |
| Q7  (multiple responses allowed and free response for “other”) | Are there any cultural reasons you consume non-alcoholic FFs and beverages?  Select all that apply.   - Heritage recipe or practice (passed down through the generations of my family) - Popular with my ethnic group - Part of the normal diet in my part of the world - Part of a culture in an area I have lived or visited - Part of a culture I enjoy experiencing - Other (please specify) - None |

**Supplementary Materials, Table 2.** Survey question block related to fermented fruits and vegetables foods.

| **Question block 2: related to all fermented fruits and vegetables** | |
| --- | --- |
| Background information  (prior to Q8) | The remainder of the survey is now focused specifically on fermented fruits and vegetables.    Examples of fermented fruits include olives (such as those found in olive bars, not cans) and pickles (fermented cucumbers), as well as preserved citrus, fermented berries, etc. Examples of fermented vegetables include sauerkraut and kimchi (fermented cabbage), and curtido (fermented cabbage relish).    This part of the survey does not include fermented alcoholic beverages like wine or beer, fermented dairy, grains, legumes, or meats.    You will also be asked questions about ready-to-eat fresh (non-thermally processed) fermented fruits and vegetables. Fresh (non-thermally processed) fermented fruits and vegetables are consumed directly after fermentation or after storage in the refrigerator. Fresh (non-thermally processed) fermented fruits and vegetables have not gone through a process to make them shelf stable by thermal processing. You can find a fresh fermented fruit or vegetables in the refrigerated section of the grocery store or sold chilled at Farmers’ Markets. Many FFs found on the shelves at room temperature in grocery stores or markets are heated-treated after fermentation. This heating process extends the shelf life of the final products while at the same time keeps the food from spoiling, but also inactivates the live microbes found in FFs.    In our survey, we want to know if you prepare and/or consume FFs that contain the live organisms responsible for fermentation. |
| **Question (Q) number and type of response allowed** | **Question text included in survey** |
| Q8  (single response allowed and free response for “yes”) | Do you currently buy fermented fruits or vegetables?   - Yes (please specify the product type and brand if you know) - No (skip to Q10) - Uncertain |
| Q9  (single response allowed) | If you buy fermented fruits and vegetable foods, do they always require refrigeration?   - Yes - No - I purchase both refrigerated and shelf-stable FFs |

**Supplementary Materials, Table 3.** Survey question block related fresh fermented fruits and vegetables.

| **Question block 3: related to fresh fermented fruits and vegetables** | |
| --- | --- |
| **Question (Q) number and type of response allowed** | **Question text included in survey** |
| Q10  (single response allowed) | How often do you consume **fresh** (non-thermally processed) **fermented** fruits or vegetables?    **Fresh fermented** fruits and vegetables are consumed directly after fermentation or after storage in the refrigerator. Fresh fermented fruits and vegetables have not gone through a process to make them shelf stable (thermal processing). You can find a fresh fermented fruit or vegetable in the refrigerated section of the grocery store or sold chilled at Farmers’ Markets.   - Daily - Weekly - Monthly - A few times a year - Never (skip to Q15) |
| Q11  (single response allowed and free response for “other”) | What is your main reason for eating fresh fermented fruits or vegetables?   - Taste - Health benefits - Cultural reasons - Other (please specify) |
| Q12  (multiple responses allowed and free response for “other”) | What flavors or aromas do you associate with fermented fruits or vegetables?  Select all that apply.   - Sourness - Umami - Sweet - Acid - Sharp - Bitter - Salty - Fizzy or bubbly - Bland - Pungent - Rancid - Other (please specify) - None |
| Q13  (multiple responses allowed and free response for “other”) | What, if any, health benefits do you associate with the consumption of fresh fermented fruits and vegetables?   - Digestive benefits - Improved gut microbiome - Probiotic - Prebiotic - Improved nutritional value - Immune system benefits - Other (please specify) - None |
| Q14  (multiple responses allowed and free response for “other”) | Are there any cultural reasons you consume non-alcoholic FFs and beverages?  Select all that apply.   - Heritage recipe or practice (passed down through the generations of my family) - Popular with my ethnic group - Part of the normal diet in my part of the world - Part of a culture in an area I have lived or visited - Part of a culture I enjoy experiencing - Other (please specify) - None |

**Supplementary Materials, Table 4.** Survey question block related to making fermented fruits and vegetables at home.

| **Question block 4: related to making fermented fruits and vegetables at home** | |
| --- | --- |
| **Question (Q) number and type of response allowed** | **Question text included in survey** |
| Q15  (single response allowed) | This part of the survey is on making fermented fruits and vegetables at home. Do you currently ferment fruits or vegetables at home?  Yes  No (skip to demographic questions) |
| Q16  (free response) | What type of fruits and vegetables do you ferment at home? (Please list) |
| Q17  (multiple responses allowed and free response for “website”, “blog posts”, and “other”) | What type of resources do you use as a source of information on how to ferment fruits and vegetables at home?   - University Extension publications (examples: National Center for Home Food Preservation, University of California Cooperative Extension Resources, Colorado State Extension, etc.) - United States Department of Agriculture (USDA) Complete Guide to Home Canning - Textbooks (examples: Microbiology and Technology of FFs, Fermentation Microbiology and Biotechnology, Principles of Fermentation Technology, etc.) - Other Books (examples: Farmhouse Culture Guide to Fermentation, NOMA guide to Fermentation, the Art of Fermentation, Fermented Vegetables, Wild Fermentation, etc.) - Website (please specify) - Blog posts (please specify) - Other (please specify) - None |
| Q18  (multiple responses allowed and free response for “other”) | What types of fruits and vegetables would you be most interested in fermenting at home that you are not making already?   - Apple - Berries (strawberry, blueberry, raspberry, etc.) - Pears - Citrus - Olives - Cranberries - Cucumbers - Peppers (including hot sauces) - Tomatoes (including tomato-based products like salsa) - Cabbage - Carrots - Onions - Leafy greens - Mixed vegetables - Other (please specify) |

**Supplementary Material, Table 5.** Survey demographic question block

| **Question block 5: demographic information** | |
| --- | --- |
| **Question (Q) number and type of response allowed** | **Question text included in survey** |
| Q19  (single response allowed) | What is your gender?   - Male - Female - Transmale - Transfemale - Non-binary/non-conforming - Prefer not to respond |
| Q20  (single response allowed) | Please identify your age range.   - 18-29 - 30-39 - 40-49 - 50-59 - 60-69 - 70 and above |
| Q21  (single response allowed) | Please identify your ethnicity.   - Hispanic or Latino(a) - Not Hispanic or Latino(a) |
| Q22  (single response allowed) | Please identify your race.   - American Indian/Alaska Native - Asian - Black/African American - Native Hawaiian/Other Pacific Islander - White - Two or more races - Other/not listed - Prefer not to state |
| Q23  (single response allowed) | Indicate your highest level of education.   - No formal degree - High school degree or diploma - Associate’s degree - Bachelor’s degree - Master’s degree - Doctoral or professional degree - Prefer not to answer |
| Q24  (single response allowed and free response for “outside the United States”) | Please share the country in which you reside.   - United States - Outside the United States (please write the country name) (skip to Q26) - Prefer not to answer |
| Q25 | Please share the state in which you reside.   - Alabama - Alaska - Arizona - Arkansas - California - Colorado - Connecticut - Delaware - Florida - Georgia - Hawaii - Idaho - Illinois - Indiana - Iowa - Kansas - Kentucky - Louisiana - Maine - Maryland - Massachusetts - Michigan - Minnesota - Mississippi - Missouri - Montana - Nebraska - Nevada - New Hampshire - New Jersey - New Mexico - New York - North Carolina - North Dakota - Ohio - Oklahoma - Oregon - Pennsylvania - Rhode Island - South Carolina - South Dakota - Tennessee - Texas - Utah - Vermont - Utah - Vermont - Virginia - Washington - West Virginia - Wisconsin - Wyoming |
| Q26  (free response) | What is your estimated annual average family income? |

**Supplementary Materials, Table 6.** Frequency of responses for Q1: “Based on this explanation and your experience, please indicate below which of the following foods can be made by fermentation or are examples of FFs and beverages. Please check all response options that apply”. Total respondents for Q1 (n=751).

| **Fermented food** | **Response Frequency (n=751)** | **Percentage** **(%)** |
| --- | --- | --- |
| Yogurt | 658 | 87.62 |
| Sour cream | 473 | 62.98 |
| Kefir | 488 | 64.98 |
| Cheese | 507 | 67.51 |
| Wine, beer, distilled spirits | 627 | 83.49 |
| Kalamata olives | 267 | 35.55 |
| Bread | 514 | 68.44 |
| Coffee | 222 | 29.56 |
| Chocolate | 255 | 34.09 |
| Kombucha | 467 | 62.18 |
| Salami | 222 | 29.56 |
| Vinegar | 323 | 43.01 |
| Kimchi | 549 | 73.10 |
| Sauerkraut | 536 | 71.37 |
| Curtido | 141 | 18.77 |
| Escabeche | 118 | 15.71 |
| Soy sauce | 349 | 46.47 |
| None of these | 0 | 0 |

**Supplementary Materials, Table 7.** Frequency of responses for Q2: “Besides alcoholic beverages (example: beer, wine, spirits), there are many other types of FFs and beverages. Do you currently consume non-alcoholic FFs and beverages? Total respondents for Q2 n=751.

| **Consumption of non-alcoholic fermented foods** | **Response (n=751)** | **Percentage (%)** |
| --- | --- | --- |
| Yes | 640 | 85.22 |
| No | 81 | 10.79 |
| Uncertain | 25 | 3.33 |

**Supplementary Materials, Table 8.** Frequency of responses for Q3: “Which types of FFs and beverages do you eat (excluding alcoholic beverages)? Please check the box next to the category of FFs and beverages you eat and indicate the frequency of consuming that food by checking box next to: daily, weekly, monthly, or a few times a year”.

| **Fermented food category** | **Consume the food** | **Reported frequency of consumption** | | | |
| --- | --- | --- | --- | --- | --- |
|  |  | **Daily** | **Weekly** | **Monthly** | **A few times a year** |
| Cereal grain | 307 | 343 | 191 | 31 | 22 |
| Fruits and vegetables | 281 | 42 | 171 | 145 | 245 |
| Dairy | 264 | 344 | 196 | 70 | 14 |
| Soybean or rice | 250 | 158 | 269 | 129 | 53 |
| Meats | 204 | 102 | 150 | 169 | 125 |
| Other* | 59 | 45 | 45 | 24 | 11 |

*Other response frequencies: Kombucha (49), chili sauce (17), vinegar (6), temeph (5), water kefir (6), chocolate (4), natto (4), fish (3), coffee (3), kvass (3), non-dairy yogurt (2), tofu (2), amazake (1), pulque (1), millet (1).

**Supplementary Materials, Table 9.** Frequency of responses for Q4: “What is your main reason for eating non-alcoholic fermented beverages?” Total respondents for Q4 n=665.

| **Reason for eating** | **Frequency** **(n=665)** | **Percent** (%) |
| --- | --- | --- |
| Taste | 337 | 50.68 |
| Health Benefits | 235 | 35.34 |
| Cultural Reasons | 80 | 12.03 |
| Other (please specify) | 13 | 1.95 |

**Supplementary Materials, Table 10.** Frequency of responses for Q5: “What flavors or aromas do you associate with FFs? Select all that apply” Total respondents for Q4 n=665.

| **Flavor/ Aroma** | **Frequency** (n=665) | **Percent** (%) |
| --- | --- | --- |
| Sourness | 540 | 81.20 |
| Umami | 310 | 46.62 |
| Sweet | 168 | 25.26 |
| Acid | 401 | 60.30 |
| Sharp | 215 | 32.33 |
| Spicy | 142 | 21.35 |
| Bitter | 57 | 8.57 |
| Salty | 236 | 35.49 |
| Fizzy or bubbly | 262 | 39.40 |
| Bland | 17 | 2.56 |
| Pungent | 176 | 26.47 |
| Rancid | 38 | 5.71 |
| Other (please specify) | 10 | 1.50 |
| None | 1 | 0.15 |

**Supplementary Materials, Table 11.** Frequency of responses for Q6: “What, if any, health benefits do you associate with non-alcoholic FFs and beverages? Select all that apply” Total respondents for Q6 n=665.

| **Health Benefit** | **Frequency (n=665)** | **Percent (%)** |
| --- | --- | --- |
| Digestive benefits | 508 | 76.39 |
| Improved gut microbiome | 513 | 77.14 |
| Probiotic | 458 | 68.87 |
| Prebiotic | 243 | 36.54 |
| Improved nutritional value | 330 | 49.62 |
| Immune system benefits | 273 | 41.05 |
| Other (please specify)  None | 21  14 | 3.16  2.11 |

**Supplementary Materials, Table 12.** Frequency of responses for Q7: “Are there any cultural reasons you consume non-alcoholic FFs and beverages? Select all that apply.” Total respondents for Q7 n=665.

| **Cultural Reasons** | **Frequency (n=665)** | **Percent (%)** |
| --- | --- | --- |
| Heritage recipe or practice (passed down through the generations) | 178 | 26.77 |
| Popular with my ethnic group | 189 | 28.42 |
| Part of the normal diet in my part of the world | 248 | 37.29 |
| Part of a culture in an area I have lived or visited | 181 | 27.22 |
| Part of a culture I enjoy experiencing | 280 | 42.11 |
| Other (please specify)  None | 16  113 | 2.41  16.99 |

**Supplementary Materials, Table 13.** Frequency of responses for Q8: “Do you currently buy fermented fruits or vegetables?” Total respondents for Q8 n=751.

| **Response** | **Frequency** (n=751) | **Percent** (%) |
| --- | --- | --- |
| Yes (please specify the product type and brand if you know) * | 398 | 53.00 |
| No  Uncertain | 267  81 | 35.55  10.79 |

*Frequency of responses to “Yes”:

| **Fermented food** | **Frequency** |  |
| --- | --- | --- |
| Kimchi | 129 |  |
| Pickles (cucumber) | 110 |  |
| Sauerkraut | 97 |  |
| Olives | 52 |  |
| Assorted vegetables | 21 |  |
| Miso | 8 |  |
| Kombucha | 7 |  |
| Yogurt | 5 |  |
| Kefir | 4 |  |
| Vinegar | 4 |  |
| Hot sauce | 4 |  |
| Radish | 3 |  |
| Citrus | 4 |  |
| Banana peppers | 3 |  |
| Garlic | 3 |  |
| Beans | 3 |  |
| Natto | 3 |  |
| Fermented berries | 2 | |
| Apple | 2 | |
| Dairy products | 2 | |
| Umeboshi plums | 2 | |
| Assorted fruits | 2 | |
| Soy sauce | 2 | |
| Green Tomatoes | 1 | |
| Weinkraut | 1 | |
| Bread | 1 | |
| Salami | 1 | |
| Sakura | 1 | |
| Coffee | 1 | |
| Chocolate | 1 | |
| Corned beef | 1 | |
| Gochujang | 1 | |

**Supplementary Materials, Table 14.** Frequency of responses for Q9: “If you buy fermented fruit and vegetable foods, do they always require refrigeration?” Total respondents for Q9 n=479.

| **Response** | **Frequency** (n=479) | **Percent** (%) |
| --- | --- | --- |
| Yes | 282 | 58.87 |
| No  I purchase both refrigerated and shelf-stable FFs | 62  135 | 12.94  28.18 |

**Supplementary Materials, Table 15.** Frequency of responses for Q10: “How often do you consume fresh (non-thermally processed) fermented fruits or vegetables?” Total respondents for Q10 n=751.

| **Response** | **Frequency** (n=751) | **Percent** (%) |
| --- | --- | --- |
| Daily  Weekly | 151  255 | 20.11  33.95 |
| Monthly  A few times a year  Never | 161  125  54 | 21.44  16.64  7.19 |

**Supplementary Materials, Table 16.** Frequency of responses for Q11: “What is your main reason for eating fresh fermented fruits or vegetables?” Total respondents for Q11 n=694.

| **Response** | **Frequency** (n=694) | **Percent** (%) |
| --- | --- | --- |
| Taste  Health benefits | 348  251 | 50.14  36.17 |
| Cultural Reasons  Other (please specify) | 71  22 | 10.23  3.17 |

**Supplementary Materials, Table 17.** Frequency of responses for Q12: “What flavors or aromas do you associate with fermented fruits or vegetables? Select all that apply.” Total respondents for Q12 n=694.

| **Response** | **Frequency** (n=694) | **Percent** (%) |
| --- | --- | --- |
| Sourness | 517 | 74.50 |
| Umami | 270 | 39.05 |
| Sweet | 190 | 27.38 |
| Acid | 378 | 54.47 |
| Sharp | 185 | 26.66 |
| Bitter | 70 | 10.09 |
| Salty | 261 | 37.61 |
| Fizzy or bubbly | 122 | 17.58 |
| Bland | 11 | 1.59 |
| Pungent | 151 | 21.76 |
| Rancid | 29 | 4.18 |
| Other (please specify) | 11 | 1.59 |
| None | 3 | 0.43 |

**Supplementary Materials, Table 18.** Frequency of responses for Q13: “What, if any, health benefits do you associate with the consumption of fresh fermented fruits and vegetables?” Total respondents for Q13 n=694.

| **Response** | **Frequency** (n=694) | **Percent** (%) |
| --- | --- | --- |
| Digestive benefits | 472 | 68.01 |
| Improved gut microbiome | 488 | 70.32 |
| Probiotic | 384 | 55.33 |
| Prebiotic | 246 | 35.45 |
| Improved nutritional value | 332 | 47.84 |
| Immune system benefits | 263 | 37.90 |
| Other (please specify) | 12 | 1.73 |
| None | 32 | 4.61 |

**Supplementary Materials, Table 19.** Frequency of responses for Q14: “Are there any cultural reasons you consume non-alcoholic FFs and beverages? Select all that apply.” Total respondents for Q14 n=694.

| **Response** | **Frequency** (n=694) | **Percent** (%) |
| --- | --- | --- |
| Heritage recipe or practice (passed down through the generations) | 159 | 22.91 |
| Popular within my ethnic group | 180 | 25.94 |
| Part of the normal diet in my area of the world | 203 | 29.25 |
| Part of a culture in an area I have lived or visited | 178 | 25.65 |
| Part of a culture I enjoy experiencing | 272 | 39.19 |
| Other (please specify) | 10 | 1.44 |
| None | 139 | 20.03 |

**Supplementary Materials, Table 20.** Frequency and percentages for association between respondents that correctly identified all FFs in Q1 and responses for Q20.

| **Age Group** | **Frequency** | **Percent** |
| --- | --- | --- |
| 18-29 | 17 | 5.63 |
| 30-39 | 18 | 7.76 |
| 40-49 | 9 | 8.65 |
| 50-59 | 1 | 2.04 |
| 60-69 | 9 | 20.93 |
| 70 and above | 1 | 6.25 |

**Supplementary Materials, Table 21.** Frequency and percentages for association between respondents that correctly identified all FFs in Q1 and responses for Q23.

| **Degree** | **Frequency** | **Percent** |
| --- | --- | --- |
| No formal degree | 1 | 5.26 |
| High school degree or diploma | 3 | 5.00 |
| Associate’s degree | 6 | 6.74 |
| Bachelor’s degree | 19 | 6.13 |
| Master’s degree | 17 | 9.24 |
| Doctoral or professional degree | 8 | 10.53 |
| Prefer not to answer | 1 | 12.5 |

**Supplementary Materials, Table 22.** Frequency and percentages for association between respondents that identified curtido and escabeche as fermented in Q1 and their response for Q21.

| **Response** | **"escabeche"** | **"escabeche" %** | **"curtido"** | **“curtido” %** |
| --- | --- | --- | --- | --- |
| Hispanic or Latino(a) | 22 | 14.01 | 30 | 19.11 |
| Not Hispanic or Latino(a) | 96 | 16.3 | 111 | 18.85 |

**Supplementary Materials, Table 23.** Frequency and percentages for association between respondents that identified kimchi as fermented in Q1 and their responses for Q22.

| **Response** | **Frequency** | **Percentage** |
| --- | --- | --- |
| American Indian/Alaska Native | 5 | 71.43 |
| Asian | 81 | 86.17 |
| Black/ African American | 14 | 70.00 |
| Native Hawaiian/ Other Pacific Islander | 8 | 53.33 |
| Other/ not listed | 12 | 66.67 |
| Prefer not to state | 20 | 80.00 |
| Two or more races | 31 | 88.57 |
| White | 376 | 70.94 |

**Supplementary Materials, Table 24.** Frequency and percentages for association between respondents that selected “cultural reasons” as their main reason for eating non-alcoholic and fresh fermented fruits and vegetables in Q4 and Q11, respectively, and responses for Q22.

| **Response** | **Frequency (Q4)** | **Percentage (Q4)** | **Frequency (Q11)** | **Percentage (Q11)** |
| --- | --- | --- | --- | --- |
| American Indian/Alaska Native | 0 | 0.00 | 1 | 14.29 |
| Asian | 9 | 9.57 | 7 | 7.45 |
| Black/ African American | 3 | 15.00 | 2 | 10.00 |
| Native Hawaiian/ Other Pacific Islander | 2 | 13.33 | 2 | 13.33 |
| Other/ not listed | 1 | 5.56 | 0 | 0.00 |
| Prefer not to state | 0 | 0.00 | 0 | 0.00 |
| Two or more races | 3 | 8.57 | 4 | 11.43 |
| White | 62 | 11.70 | 55 | 10.38 |

**Supplementary Materials, Table 25**. Comparison between responses to Q6 with responses to Q3 for the three most selected health benefits associated with FFs: probiotic, improved gut microbiome, and digestive benefits.

| **Reason (Q6)** | **Category (Q3)** | **Consumption (Q3)** | **Frequency (Q3)** |
| --- | --- | --- | --- |
| probiotic | cereal grain | A few times a year | 19 |
| probiotic | cereal grain | Daily | 224 |
| probiotic | cereal grain | I eat this food | 43 |
| probiotic | cereal grain | Monthly | 21 |
| probiotic | cereal grain | Weekly | 138 |
| probiotic | fruits and vegetables | A few times a year | 33 |
| probiotic | fruits and vegetables | Daily | 117 |
| probiotic | fruits and vegetables | I eat this food | 27 |
| probiotic | fruits and vegetables | Monthly | 99 |
| probiotic | fruits and vegetables | Weekly | 167 |
| probiotic | dairy | A few times a year | 10 |
| probiotic | dairy | Daily | 226 |
| probiotic | dairy | I eat this food | 20 |
| probiotic | dairy | Monthly | 44 |
| probiotic | dairy | Weekly | 142 |
| probiotic | soybean | A few times a year | 39 |
| probiotic | soybean | Daily | 99 |
| probiotic | soybean | I eat this food | 20 |
| probiotic | soybean | Monthly | 92 |
| probiotic | soybean | Weekly | 189 |
| probiotic | meats | A few times a year | 95 |
| probiotic | meats | Daily | 57 |
| probiotic | meats | I eat this food | 16 |
| probiotic | meats | Monthly | 121 |
| probiotic | meats | Weekly | 102 |
| probiotic | other | A few times a year | 4 |
| probiotic | other | Daily | 33 |
| probiotic | other | I eat this food | 3 |
| probiotic | other | Monthly | 19 |
| probiotic | other | Weekly | 31 |
| improved gut microbiome | cereal grain | A few times a year | 18 |
| improved gut microbiome | cereal grain | Daily | 247 |
| improved gut microbiome | cereal grain | I eat this food | 43 |
| improved gut microbiome | cereal grain | Monthly | 23 |
| improved gut microbiome | cereal grain | Weekly | 168 |
| improved gut microbiome | fruits and vegetables | A few times a year | 38 |
| improved gut microbiome | fruits and vegetables | Daily | 131 |
| improved gut microbiome | fruits and vegetables | I eat this food | 37 |
| improved gut microbiome | fruits and vegetables | Monthly | 103 |
| improved gut microbiome | fruits and vegetables | Weekly | 190 |
| improved gut microbiome | dairy | A few times a year | 14 |
| improved gut microbiome | dairy | Daily | 271 |
| improved gut microbiome | dairy | I eat this food | 17 |
| improved gut microbiome | dairy | Monthly | 49 |
| improved gut microbiome | dairy | Weekly | 146 |
| improved gut microbiome | soybean | A few times a year | 44 |
| improved gut microbiome | soybean | Daily | 108 |
| improved gut microbiome | soybean | I eat this food | 25 |
| improved gut microbiome | soybean | Monthly | 96 |
| improved gut microbiome | soybean | Weekly | 219 |
| improved gut microbiome | meats | A few times a year | 109 |
| improved gut microbiome | meats | Daily | 61 |
| improved gut microbiome | meats | I eat this food | 22 |
| improved gut microbiome | meats | Monthly | 122 |
| improved gut microbiome | meats | Weekly | 114 |
| improved gut microbiome | other | A few times a year | 10 |
| improved gut microbiome | other | Daily | 36 |
| improved gut microbiome | other | I eat this food | 6 |
| improved gut microbiome | other | Monthly | 18 |
| improved gut microbiome | other | Weekly | 41 |
| digestive benefits | cereal grain | A few times a year | 21 |
| digestive benefits | cereal grain | Daily | 257 |
| digestive benefits | cereal grain | I eat this food | 33 |
| digestive benefits | cereal grain | Monthly | 27 |
| digestive benefits | cereal grain | Weekly | 156 |
| digestive benefits | fruits and vegetables | A few times a year | 38 |
| digestive benefits | fruits and vegetables | Daily | 123 |
| digestive benefits | fruits and vegetables | I eat this food | 33 |
| digestive benefits | fruits and vegetables | Monthly | 119 |
| digestive benefits | fruits and vegetables | Weekly | 181 |
| digestive benefits | dairy | A few times a year | 12 |
| digestive benefits | dairy | Daily | 253 |
| digestive benefits | dairy | I eat this food | 11 |
| digestive benefits | dairy | Monthly | 54 |
| digestive benefits | dairy | Weekly | 164 |
| digestive benefits | soybean | A few times a year | 41 |
| digestive benefits | soybean | Daily | 113 |
| digestive benefits | soybean | I eat this food | 20 |
| digestive benefits | soybean | Monthly | 99 |
| digestive benefits | soybean | Weekly | 216 |
| digestive benefits | meats | A few times a year | 110 |
| digestive benefits | meats | Daily | 57 |
| digestive benefits | meats | I eat this food | 18 |
| digestive benefits | meats | Monthly | 130 |
| digestive benefits | meats | Weekly | 114 |
| digestive benefits | other | A few times a year | 8 |
| digestive benefits | other | Daily | 40 |
| digestive benefits | other | I eat this food | 6 |
| digestive benefits | other | Monthly | 16 |
| digestive benefits | other | Weekly | 39 |

**Supplementary Materials, Table 26.** Frequency of responses for Q15: “This part of the survey is on making fermented fruits and vegetables at home. Do you currently ferment fruits or vegetables at home?” Total respondents for Q15 n=746.

| **Ferment fruits or vegetables at home** | **Response** (n=746) | **Percentage (%)** |
| --- | --- | --- |
| Yes | 367 | 49.20 |
| No | 379 | 50.80 |

**Supplementary Materials, Table 27.** Frequency of responses for Q16: “What type of fruits and vegetables do you ferment at home? (Please list)” Total respondents for Q16 n=367.

| **Fruit or vegetable fermented at home** | **Response Frequency** (n=367) |
| --- | --- |
| Cabbage | 202 |
| Cucumber | 88 |
| Alliums | 54 |
| Carrot | 52 |
| Peppers | 48 |
| Citrus | 45 |
| Radish | 40 |
| Apple | 22 |
| Leafy greens | 22 |
| Tomatoes | 18 |
| Beets | 17 |
| Pineapple | 16 |
| Berries | 15 |
| Stone fruit | 15 |
| Grape | 13 |
| Kombucha | 11 |
| Assorted vegetables | 11 |
| Assorted fruit | 9 |
| Olives | 9 |
| Turnip | 8 |
| Asparagus | 6 |
| Bread | 6 |
| Cauliflower | 6 |
| Ginger | 6 |
| Green beans | 5 |
| Vinegar | 5 |
| Kiwi | 4 |
| Mango | 4 |
| Beans | 3 |
| Passion fruit | 3 |
| Pear | 3 |
| Potatoes | 3 |
| Salsa | 3 |
| Avocado | 2 |
| Bamboo shoot | 2 |
| Eggplant | 2 |
| Kvass | 2 |
| Water kefir | 2 |

**Supplementary Materials, Table 28.** Frequency of responses for Q17: “What type of resources do you use as a source of information on how to ferment fruits and vegetables at home?” Total respondents for Q15 n=367.

| **Resource type** | **Response** (n=367) | **Percentage (%)** |
| --- | --- | --- |
| University Extension publications (examples: National Center for Home Food Preservation, University of California Cooperative Extension Resources, Colorado State Extension, etc.) | 89 | 24.25 |
| United States Department of Agriculture (USDA) Complete Guide to Home Canning | 91 | 24.80 |
| Textbooks (examples: Microbiology and Technology of FFs, Fermentation Microbiology and Biotechnology, Principles of Fermentation Technology, etc.) | 115 | 31.34 |
| Other Books (examples: Farmhouse Culture Guide to Fermentation, NOMA guide to Fermentation, the Art of Fermentation, Fermented Vegetables, Wild Fermentation, etc.) | 170 | 46.32 |
| Website (please specify) | 51 | 23.90 |
| YouTube | 142 | 38.69 |
| Blog posts (please specify) | 21 | 5.72 |
| Other (specified below) | 50 | 13.62 |
| Community knowledge | 28 |  |
| Personal knowledge | 7 |  |
| Social media | 2 |  |
| None | 12 | 3.27 |

**Supplementary Materials, Table 29.** Frequency of responses for Q18: “What types of fruits and vegetables would you be most interested in fermenting at home that you are not making already?” Total respondents for Q18 n=367.

| **Ferment fruits or vegetables at home** | **Response (n=367)** | **Percentage (%)** |
| --- | --- | --- |
| Apple | 88 | 23.98 |
| Berries (strawberry, blueberry, raspberry, etc.) | 107 | 29.16 |
| Pears | 69 | 18.80 |
| Citrus | 89 | 24.25 |
| Olives | 104 | 28.33 |
| Cranberries | 54 | 14.71 |
| Cucumbers | 138 | 37.60 |
| Peppers (including hot sauces) | 105 | 28.61 |
| Tomatoes (including tomato-based products like salsa) | 95 | 25.89 |
| Cabbage | 97 | 26.43 |
| Carrots | 86 | 23.43 |
| Onions | 62 | 16.89 |
| Leafy greens | 68 | 18.53 |
| Mixed vegetables | 75 | 20.44 |
| Other (specified below) | 22 | 5.99 |
| Plant-based cheese | 3 |  |
| Stone fruit | 2 |  |
| Miso | 2 |  |
| Eggplant | 1 |  |
| Squash | 1 |  |
| Radish | 1 |  |
| Yuzu | 1 |  |
| Edible flowers | 1 |  |
| Beans | 1 |  |
| Beets | 1 |  |
| Mushrooms | 1 |  |

**Supplementary Materials, Table 30.** Comparison between “Yes” responses to Q15 (“Do you currently ferment fruits or vegetables at home?”) and Q23 (“Indicate your highest level of education”).

| **Education Level** | **Frequency** | **Percentage** |
| --- | --- | --- |
| No formal degree | 13 | 68.42 |
| High school degree or diploma | 29 | 48.33 |
| Associate’s degree | 46 | 51.69 |
| Bachelor’s degree | 149 | 48.07 |
| Master’s degree | 87 | 47.28 |
| Doctoral or professional degree | 37 | 48.68 |
| Prefer not to answer | 6 | 75.00 |

**Supplementary Materials, Table 31.** Comparison between “Yes” responses to Q15 (“Do you currently ferment fruits or vegetables at home?”) and Q20(“Please identify your age range”).

| **Age** | **Frequency** | **Percentage** |
| --- | --- | --- |
| 18-29 | 91 | 30.13 |
| 30-39 | 132 | 56.90 |
| 40-49 | 70 | 67.31 |
| 50-59 | 31 | 63.27 |
| 60-69 | 31 | 72.09 |
| 70 and above | 12 | 75.00 |

**Supplementary Materials, Table 32.** Frequency of free responses for Q24: “Please share the country in which you reside.” Total respondents for Q24 n=751.

| **Country Outside the United States** | **Response (n=751)** |
| --- | --- |
| Canada | 11 |
| Argentina | 10 |
| Argentina | 8 |
| United Kingdom | 8 |
| Italy | 7 |
| Peru | 3 |
| Switzerland | 3 |
| Brazil | 3 |
| India | 3 |
| Korea | 2 |
| Germany | 2 |
| Mexico | 2 |
| Japan | 2 |
| India | 2 |
| Chile | 1 |
| Qatar | 1 |
| France | 1 |
| Ireland | 1 |
| Taiwan | 1 |
| Ireland | 1 |
| Netherlands | 1 |
| China | 1 |
| Philippines | 1 |
| Colombia | 1 |
| Australia | 1 |
| Czechia | 1 |
| Belize | 1 |
| Latvia | 1 |
| Estonia | 1 |

**Supplementary Materials, Table 33**. Frequency of responses for Q25: “Please share the state in which you reside.” Total respondents for Q25 n=751.

| **State** | **Frequency (n=751)** | **Percentage (%)** |
| --- | --- | --- |
| Alabama | 13 | 1.73 |
| Alaska | 20 | 2.66 |
| Arizona | 16 | 2.13 |
| Arkansas | 11 | 1.46 |
| California | 314 | 41.81 |
| Colorado | 13 | 1.73 |
| Connecticut | 10 | 1.33 |
| Delaware | 7 | 0.93 |
| Florida | 22 | 2.93 |
| Georgia | 11 | 1.46 |
| Hawaii | 9 | 1.20 |
| Idaho | 7 | 0.93 |
| Illinois | 9 | 1.20 |
| Indiana | 5 | 0.67 |
| Iowa | 7 | 0.93 |
| Kansas | 15 | 2.00 |
| Kentucky | 5 | 0.67 |
| Louisiana | 3 | 0.40 |
| Maine | 2 | 0.27 |
| Maryland | 3 | 0.40 |
| Massachusetts | 4 | 0.53 |
| Michigan | 11 | 1.46 |
| Minnesota | 7 | 0.93 |
| Mississippi | 2 | 0.27 |
| Missouri | 3 | 0.40 |
| Montana | 1 | 0.13 |
| Nebraska | 5 | 0.67 |
| Nevada | 3 | 0.40 |
| New Hampshire | 1 | 0.13 |
| New Jersey | 2 | 0.27 |
| New Mexico | 1 | 0.13 |
| New York | 18 | 2.40 |
| North Carolina | 12 | 1.60 |
| North Dakota | 1 | 0.13 |
| Ohio | 4 | 0.53 |
| Oklahoma | 3 | 0.40 |
| Oregon | 11 | 1.46 |
| Pennsylvania | 6 | 0.80 |
| Rhode Island | 2 | 0.27 |
| South Carolina | 2 | 0.27 |
| South Dakota | 1 | 0.13 |
| Tennessee | 7 | 0.93 |
| Texas | 14 | 1.86 |
| Utah | 4 | 0.53 |
| Vermont | 2 | 0.27 |
| Virginia | 5 | 0.67 |
| Washington | 15 | 2.00 |
| West Virginia | 9 | 1.20 |
| Wisconsin | 2 | 0.27 |
| Wyoming | 1 | 0.13 |
